# Synthesis of New 3-Heteroarylindoles as Potential Anticancer Agents

**DOI:** 10.3390/molecules21070929

**Published:** 2016-07-16

**Authors:** Abdou O. Abdelhamid, Sobhi M. Gomha, Nadia A. Abdelriheem, Saher M. Kandeel

**Affiliations:** 1 Department of Chemistry, Faculty of Science, Cairo University, Giza 12613, Egypt; abdelhamid45@gmail.com (A.O.A.); nadia.abdelhamid5@gmail.com (N.A.A.); 2 Chemistry of Natural Compounds, Department National Research Center, Dokki 12622, Egypt; SaherKamel@yahoo.com

**Keywords:** thiazoles, pyrazoles, coupling reactions, thiosemicarbazides, molecular docking, anti-cancer activity

## Abstract

2-(3-(1*H*-Indol-3-yl)-5-(*p*-tolyl)-4,5-dihydro-1*H*-pyrazol-1-yl)-4-substituted-5-(substituted diazenyl)thiazoles and 2-(1*H*-indol-3-yl)-9-substituted-4,7-disubstituted pyrido[3,2-*e*][1,2,4]triazolo[4,3-*a*]pyrimidin-5(7*H*)-ones were synthesized via reaction of hydrazonoyl halides with each of 3-(1*H*-indol-2-yl)-5-(*p*-tolyl)-4,5-dihydro-1*H*-pyrazole-1-carbothioamide and 7-(1*H*-indol-3-yl)-2- thioxo-5-substituted-2,3-dihydropyrido[2,3-*d*]pyrimidin-4(1*H*)-ones, respectively. Also, hydrazonoyl halides were reacted with *N*’-(1-(1*H*-indol-3-yl)ethylidene)-2-cyanoacetohydrazide to afford 1,3,4-thiadiazole derivatives. Structures of the new synthesis were elucidated on the basis of elemental analysis, spectral data, and alternative synthetic routes whenever possible. Fifteen of the new compounds have been evaluated for their antitumor activity against the MCF-7 human breast carcinoma cell line. The results indicated that many of the tested compounds showed moderate to high anticancer activity when compared with doxorubicin as a reference drug.

## 1. Introduction

**Figure 1:**
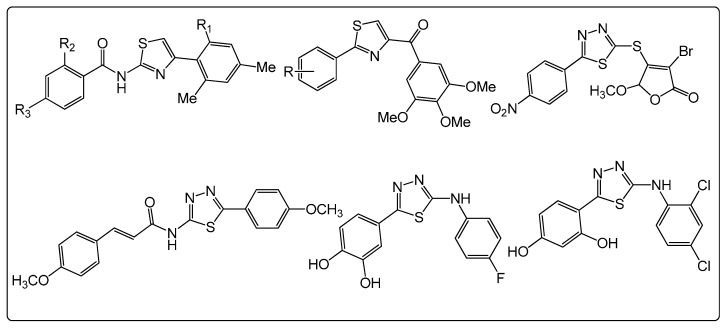
Lead compounds among thiazole and thiadiazole derivatives with anticancer activity.

The wide-ranging biological activity associated with indole derivatives, both naturally occurring and synthetic, ensures that the synthesis of indole derivatives remains a topic of current interest [1,2,3,4]. Monoindole and bisindole have been intensively studied and the results revealed that most of them have biological activities; for example, indole-3-carbinol, found in Brassica plants, is a potential cancer protective agent [5,6]. Thiazoles can be found in drug development for the treatment of allergies [7], hypertension [8], inflammation [9], schizophrenia [10], bacterial infections [11], HIV [12], sleep disorders [13], and for the treatment of pain [14], as fibrinogen receptor antagonists with antithrombotic activity [15] and as new inhibitors of bacterial DNA gyrase B [16]. The 1,2,4-triazolopyrimidines have also attracted growing interest due to their important pharmacological activities, such as antitumor, antimalarial, antimicrobial, anti-inflammatory, and antifungal activity, as well as macrophage activation [17,18,19,20,21,22]. 1,3,4-Thiadiazole derivatives have attracted considerable interest owing to their wide ranging biological activities such as antibacterial, antifungal, antituberculosis, antihepatitis B viral, antileishmanial, anti-inflammatory, analgesic, CNS depressant, anticancer, antioxidant, antidiabetic, molluscicidal, antihypertensive, diuretic, analgesic, antimicrobial, antitubercular, and anticonvulsant activities [23,24,25,26,27,28,29,30,31,32]. In light of these facts, we have synthesized some new thiazole, dihydropyrido[3,2-*e*][1,2,4]triazolo[4,3-*a*]pyrimidine and 1,3,4-thiadiazole derivatives using 3-acetylindole as a common precursor and screened these compounds for their anticancer activities. A literature survey showed that many derivatives of thiazole and 1,3,4-thiadiazole have antitumor activity with excellent IG_50_ and IC_50_ as depicted in Figure 1 [33,34,35,36,37]. In view of these facts, we report herein the synthesis of a new series of thiazoles and 1,3,4-thiadiazoles bearing indole moiety for the examination of their antitumor activity against the MCF-7 human breast carcinoma cell line.

## 2. Results and Discussion

### 2.1. Chemistry

**Scheme 1:**
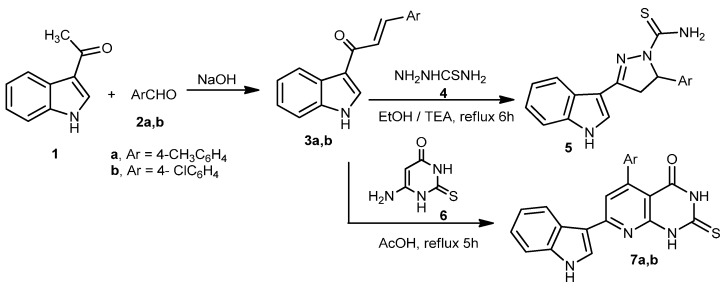
Synthesis of pyrazole-1-carbothioamide **5** and pyrido[2,3-*d*]pyrimidinthione derivatives **7a**,**b**.

1-(1*H*-Indol-3-yl)-3-(*p*-tolyl)prop-2-en-1-one (**3a**) [38] and 3-(4-chlorophenyl)-1-(1*H*-indol-3-yl) prop-2-en-1-one (**3b**) [39] were prepared as reported in the literature from reaction of 3-acetylindole (**1**) with *p*-tolualdehyde (**2a**) and 4-chlorobenzaldehyde (**2b**), respectively (Scheme 1).

Treatment of 3-aryl-1-(1*H*-indol-3-yl)prop-2-en-1-ones **3a**,**b** with thiosemicarbazide (**4**) afforded 3-(1*H*-indol-2-yl)-5-(*p*-tolyl)-4,5-dihydro-1*H*-pyrazole-1-carbothioamide (**5**). Similar treatment with 6-amino-2-thioxo-2,3-dihydropyrimidin-4(1*H*)-one (**6**) generated 5-aryl-7-(1*H*-indol-3-yl)-2-thioxo- 2,3-dihydropyrido[2,3-*d*]pyrimidin-4(1*H*)-ones **7a**,**b**, respectively (Scheme 1). Structures **5** and **7** were elucidated by elemental analysis, spectral data, and chemical transformation.

**Scheme 2:**
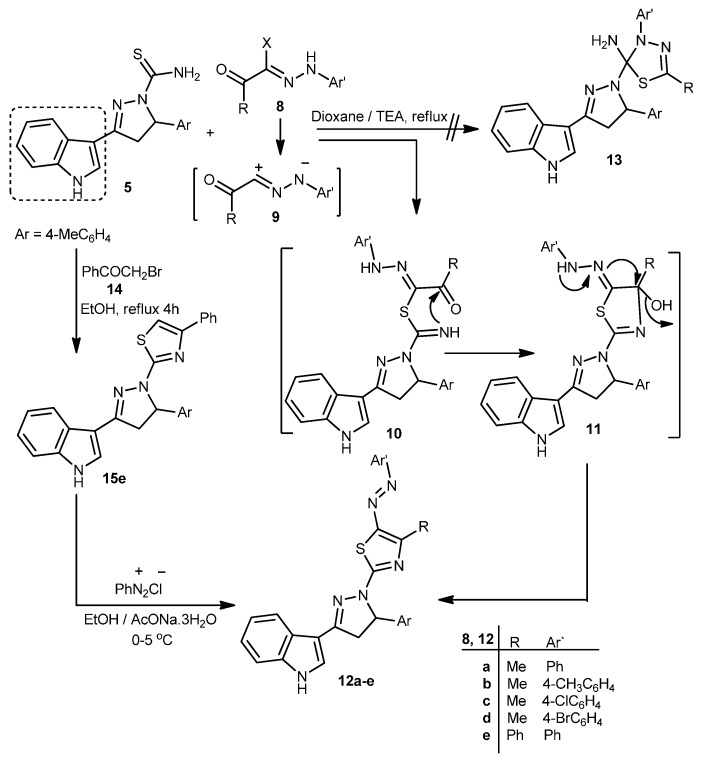
Synthesis of arylazothiazole derivatives **12a**–**e**.

Compound **5** was reacted with the appropriate keto-hydrazonoyl halides **8a**–**e** in dioxane containing a catalytic amount of TEA, to afford 2-(3-(1*H*-indol-3-yl)-5-(*p*-tolyl)-4,5-dihydro- 1*H*-pyrazol-1-yl)-5-(aryldiazenyl)-4-substituted thiazoles **12a**–**e**. Coupling of 2-(3-(1*H*-indol-3-yl)-5- (*p*-tolyl)-4,5-dihydro-1*H*-pyrazol-1-yl)-4-phenylthiazole (**15e**)[prepared by reaction of **5** with phenacyl bromide (**14**)] with benzenediazonium chloride in ethanolic sodium acetate solution at 0 °C afforded a product identical to **12e** in all aspects (mp, mixed mp, and spectra). In light of these results, the mechanism outlined in Scheme 2 seems to be the most plausible pathway for the formation of **12a** from the reaction of **5** with **8a**. The reaction involves initial formation of thiohydrazonate **10**, which undergoes cyclization as soon as it is formed to yield the intermediate **11**. The latter loses one molecule of water to give final product **12a**. Alternatively, 1,3-dipolar cycloaddition of nitrilimine **9a** [prepared in situ from **8a** with triethylamine] to the C=S double bond of **5** could also lead to **10**. The formation of **11** and **12** are similar to the previously reported reactions of hydrazonoyl chloride with 1-phenyl-1,4-dihydrotetrazole-5-thione [40] and 5-phenyl-1,3,4- thiadiazole-2(3*H*)-thione [41]. Another possible product, 1-(5-(3-(1*H*-indol-3-yl)-5-(*p*-tolyl) -4,5-dihydro-1*H*-pyrazol-1-yl)-5-amino-4-phenyl-4,5-dihydro-1,3,4-thiadiazol-2-yl)ethan-1-one (**13**), was ruled out by elemental analysis and spectral data. Analogously, treatment of the appropriate **5** with each of **8b**–**e** gave 2-(3-(1*H*-indol-3-yl)-5-(*p*-tolyl)-4,5-dihydro-1*H*-pyrazol-1-yl)-4- substituted-5-(substituted-diazenyl)thiazoles **12b**–**e**, respectively, in good yield (Scheme 2).

**Scheme 3:**
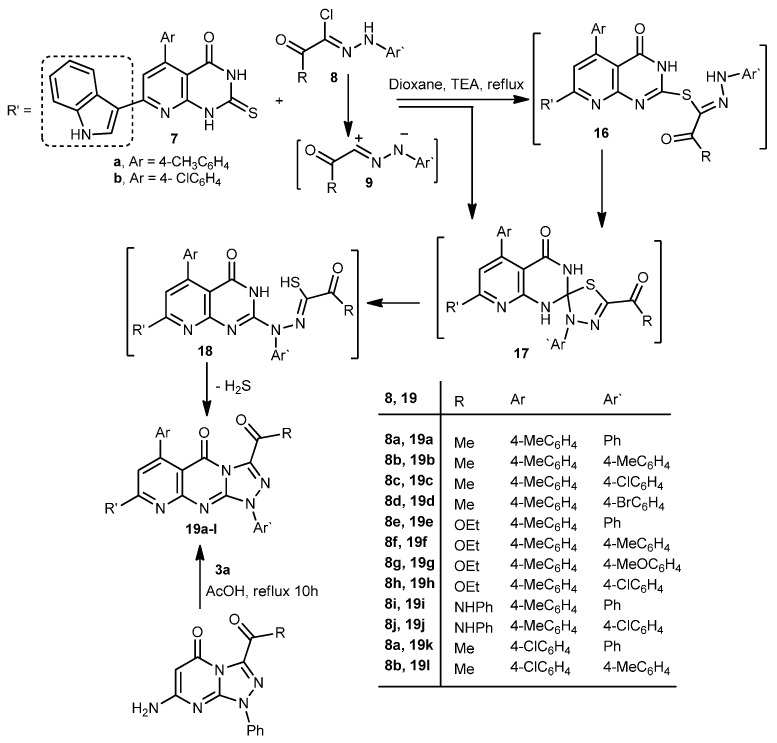
Synthesis of triazolopyridopyrimidinones **19a**–**l**.

Treatment of the appropriate 5-aryl-7-(1*H*-indol-3-yl)-2-thioxo-2,3-dihydropyrido[2,3-*d*] pyrimidin-4(1*H*)-ones **7a** or **7b** with the appropriate hydrazonoyl halides **8a**–**j** in dioxane containing TEA under reflux gave 2-(1*H*-indol-3-yl)-9-substituted-4,7-disubstituted pyrido[3,2-*e*] [1,2,4]triazolo[4,3-*a*]pyrimidin-5(7*H*)-ones **19a**–**l**, respectively (Scheme 3). Structure **19** was elucidated by elemental analysis, spectral data, and alternative synthetic routes. Thus, treatment of 7-amino-3-substituted-1-phenyl-[1,2,4]triazolo[4,3-*a*]pyrimidin-5(1*H*)-ones **20a**,**e**,**i** [42] with chalcone **3a** in boiling acetic acid made products identical in all respects (mp., mixed mp., and spectra) with the corresponding **19a**,**e**,**i**.

The mechanism in Scheme 3 outlines what seems to be the most plausible pathway for the formation of **19** from the reaction of thione **7** with **8** via two pathways. In the first, 1,3-addition of the thiol tautomer **7** to the nitrilimine **9** would give the thiohydrazonate ester **16** which would undergo nucleophilic cyclization to yield spiro compounds **17**. Ring opening to give **18** followed by cyclization with loss of hydrogen sulfide would then yield **19**. In the second pathway, an initial 1,3-cycloaddition of nitrilimine **9** to the C=S double bond of **7** would give **17** directly (Scheme 3). Attempts to isolate the thiohydrazonate ester **16**, spiro intermediate **17** and thiohydrazide **18** did not succeed, even under mild conditions as they readily undergo in situ cyclization followed by elimination of hydrogen sulfide to give the final product **19**. This structural assignment is also consistent with literature reports, which indicate that reaction of hydrazonoyl halides with 2-thioxo-pyrimidin-4-one yielded regioselectively the corresponding 1,2,4-triazolo[4,3-*a*]pyrimidin- 5-one derivatives [42].

**Scheme 4:**
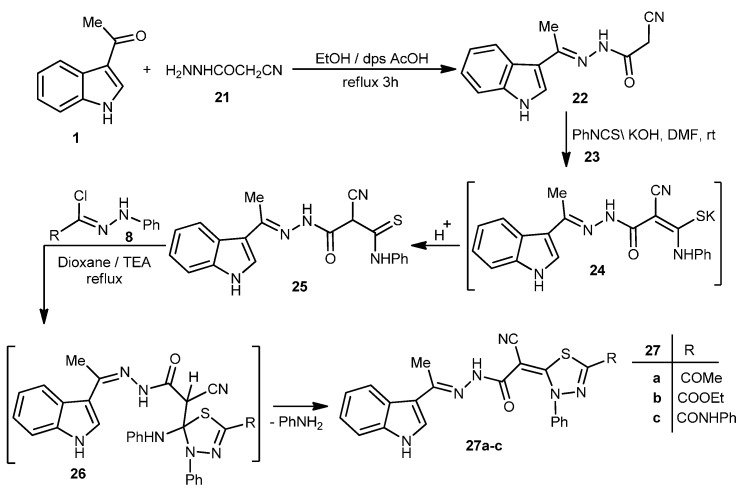
Synthesis of thiadiazoles **27a**–**c**.

Finally, 2-cyanoacetohydrazide (**21**) was reacted with 3-acetylindole (**1**) in boiling ethanol containing a catalytic amount of acetic acid to afford *N*’-(1-(1*H*-indol-3-yl) ethylidene)-2-cyanoacetohydrazide (**22**) in good yield (Scheme 4). Structure **22** was elucidated by elemental analysis, spectral data, and chemical transformation. Treatment of **22** with phenylisothiocyanate (**23**) in DMF in the presence of potassium hydroxide at room temperature followed by acid work-up yielded the thioanilide **25**. Refluxing thioanilide 25 with the appropriate hydrazonoyl chlorides **8a**,**e**,**i** in ethanolic triethylamine, gave the respective 1,3,4-thiadiazoles **27a**–**c**. (Scheme 4). Thiadiazoles **27a**–**c** were elucidated by elemental analysis and spectral data.

### 2.2. Biological Screening (Cytotoxic Activity)

The in vitro growth inhibitory rates (%) and inhibitory growth activity (as measured by IC_50_) of the newly synthesized compounds were determined against the MCF-7 human breast carcinoma cell line in comparison with the well-known anticancer drug doxorubicin as the standard, using the MTT viability assay. Data generated were used to plot a dose response curve from which the concentration (μM) of test compounds required to kill 50% of cell population (IC_50_) was determined. Cytotoxic activity was expressed as the mean IC_50_ of three independent experiments. The difference between inhibitory activities of all compounds with different concentrations was statistically significant *p* < 0.001.

**Figure 2:**
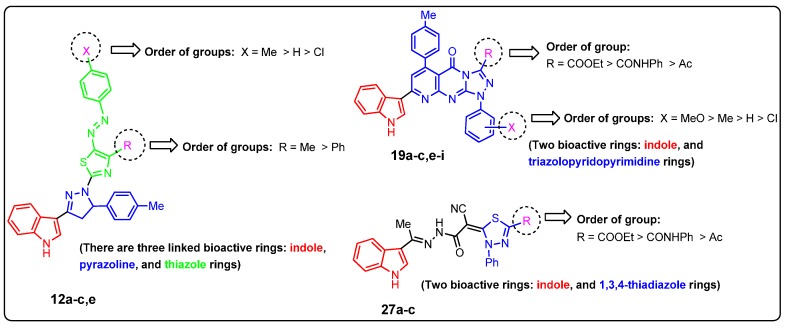
Activities of tested compounds against the MCF-7 breast cancer cell line.

The results revealed that the tested compounds showed high variation in the inhibitory growth rates and activities against the tested tumor cell lines in a concentration dependent manner compared to the reference drug as shown in Table 1 and Figure 2.

The descending order of activity of the newly synthesized compounds was as follows: **12b** > **27b** > **27c** > **27a** > **19g** > **19b** > **19e** > **12a** > **19f** > **19i** > **19a** > **12c** > **19h** > **12e** > **19c**.

### 2.3. Examination of the Compound Activities Leads to the Following Conclusions

- The activities of the synthesized compounds depend on the structural skeleton and electronic environment of the molecules.
- Based on our limited study, the 1,3,4-thiadiazole ring as in **27** has in vitro inhibitory activity greater than the 1,3-thiazole ring in **12** and more than the triazolopyridopyrimidine ring in **19**.

#### 2.3.1. For the 1,3-Thiazole Ring **12a**–**c**,**e**

- The in vitro inhibitory activity of the 4-methylthiazole is greater than 4-phenylthiazole (**12a** > **12e**). This may be due to the positive inductive effect (+I effect) of the methyl group (increase activity) or the steric effect caused by phenyl group (decrease activity).
- The introduction of electron-donating group (methyl) at C4 of the phenyl group at position 4 in the 1,3-thiazole ring enhances the antitumor activity. In contrast, introduction of an electron-withdrawing group (chlorine) decreases the antitumor activity (**12b** > **12a** > **12c**).

#### 2.3.2. For Triazolopyridopyrimidines **19a**–**c**,**e**–**i**

- For substituent at position 3: the ester group (CO_2_Et) gives higher activity than the amide group (CONHPh) or the acetyl group (Ac) (**19e** > **19i** > **19a**).
- Generally, on fixing the substituents at position 3, the electron-donating group (methyl or methoxy) at C4 of the phenyl ring enhances the antitumor activity while the electron-withdrawing group (chlorine) decreases the antitumor activity (**19b** > **19a** > **19c** and **19g** > **19f** > **19e** > **19h**).

#### 2.3.3. For 1,3,4-Thiadiazoles **27a**–**c**

- The in vitro inhibitory activity of compounds with substituents at position 5 are in the order of: COOEt > CONHPh > CH_3_CO (**27b** > **27c** > **27a**).

## 3. Experimental

### 3.1. Chemistry

All melting points were measured on an Electro thermal IA 9000 series digital melting point apparatus (Bibby Sci. Lim. Stone, Staffordshire, UK). The IR spectra were recorded on potassium bromide discs using a PyeUnicam SP 3300 or Shimadzu FT IR 8101 PC infrared spectrophotometer (Shimadzu, Tokyo, Japan). The NMR Spectra were recorded at 270 MHz on a Varian Mercury VX-300 NMR spectrometer (Varian, Inc., Karlsruhe, Germany). NMR spectra were recorded on a Varian Mercury VX-300 NMR spectrometer (Bruker BioSpin GmbH, Rheinstetten, Germany) operating at 300 MHz (^1^H-NMR) and run in deuterated dimethylsulfoxide (DMSO-*d*_6_). Chemical shifts were related to that of the solvent. ^13^C-NMR spectra were recorded at 75 MHz. Mass spectra were recorded on a Shimadzu GCMS-QP1000 EX mass spectrometer (Tokyo, Japan) at 70 eV. Elemental analyses and the biological evaluation of the products were carried out at the Microanalytical Centre of Cairo University, Giza, Egypt. All reactions were followed by TLC (silica gel coated aluminum sheets 60 F254, Merck, Merck & Co., Inc., Kenilworth, NJ, USA). Hydrazonoyl halides were prepared as reported in the literature [43,44,45,46].

#### 3.1.1. Synthesis of 3-(1*H*-Indol-3-yl)-5-(*p*-tolyl)-4,5-dihydro-1*H*-pyrazole-1-carbothioamide (**5**)

To a mixture of 1-(1*H*-indol-3-yl)-3-(*p*-tolyl)prop-2-en-1-one (**3a**) (2.61 g, 10 mmol) and thiosemicarbazide (0.92 g, 10 mmol) in EtOH (20 mL), a catalytic amount of triethylamine (1 mL) was added, then heated under reflux for 6 h. The resulting solid was collected, washed with EtOH and recrystallized from acetic acid to give pure **5** as white solid (74%); mp 179–181 °C; IR (KBr): *v* 3426, 3212, 3175 (NH_2_ and NH), 1569 (C=N) cm^−1^; ^1^H-NMR (DMSO-*d*_6_): δ 2.35 (s, 3H, CH_3_), 3.17 (dd, 1H, H_A_, *J* = 17.2, 6.3 Hz), 3.46 (dd, 1H, H_B_, *J* = 17.2, 12.1 Hz), 5.87 (dd, 1H, H_X_, *J* = 12.2, 6.3 Hz), 7.02–8.34 (m, 8H, Ar-H), 8.72 (s, 1H, Indole-H_2_), 11.54 (br s, 2H, NH_2_), 12.10 (br s, 1H, NH); MS *m*/*z* (%): 334 (M^+^, 11), 228 (43), 196 (48), 109 (100), 79 (29), 52 (27). Anal. Calcd. for C_19_H_18_N_4_S (334.44): C, 68.23; H, 5.42; N, 16.75. Found C, 68.18; H, 5.35; N, 16.59.

#### 3.1.2. Synthesis of Thiones (**7a**,**b**)

A mixture of chalcones **3a**,**b** (10 mmol) and 6-amino-2-thioxo-2,3,4-trihydro-1*H*- pyrimidin-4-one (**6**) (1.43 g, 10 mmol) in glacial acetic acid (30 mL) was heated under reflux for 5 h. After cooling, the reaction mixture was poured into an ice/conc HCl mixture and the formed solid was collected and recrystallized from DMF to give thiones **7a**,**b**, respectively.

*7-(1H-Indol-3-yl)-2-thioxo-5-(p-tolyl)-2,3-dihydropyrido[2,3-d]pyrimidin-4(1H)-one* (**7a**). Yellow crystals, 72%, mp 265–267 °C; IR (KBr): *v* 3442, 3392, 3271 (3NH), 1675 (C=O), 1595 (C=N) cm^−1^; ^1^H-NMR (DMSO-*d*_6_) : δ 2.44 (s, 3H, CH_3_), 4.69 (br s, 1H, NH), 7.15–8.28 (m, 9H, Ar-H and pyridine-H), 8.69 (s, 1H, indole-H_2_), 11.46 (br s, 1H, NH), 11.88 (br s, 1H, NH); MS, *m*/*z* (%) 384 (M^+^, 23), 249 (68), 192 (100), 119 (26), 73 (83). Anal. Calcd. for C_22_H_16_N_4_OS (384.45): C, 68.73; H, 4.19; N, 14.57. Found: C, 68.59; H, 4.06; N, 14.43.

*5-(4-Chlorophenyl)-7-(1H-indol-3-yl)-2-thioxo-2,3-dihydropyrido[2,3-d]pyrimidin-4(1H)-one* (**7b**). Yellow crystals, 76%, mp 276–278 °C; IR (KBr): *v* 3463, 3368, 3163 (3NH), 1678 (C=O), 1595 (C=N) cm^−1^; ^1^H-NMR (DMSO-*d*_6_): δ 2.44 (s, 3H, CH_3_), 4.70 (br s, 1H, NH), 7.10–8.32 (m, 9H, Ar-H and pyridine-H), 8.72 (s, 1H, indole-H_2_), 11.48 (br s, 1H, NH), 12.04 (br s, 1H, NH); MS, *m*/*z* (%) 404 (M^+^, 3), 278 (100), 151 (66), 105 (18), 57 (23). Anal. Calcd. for C_21_H_13_ClN_4_OS (404.87): C, 62.30; H, 3.24; N, 13.84. Found: C, 62.18; H, 3.20; N, 13.65.

#### 3.1.3. Synthesis of 2-(3-(1*H*-Indol-3-yl)-5-(*p*-tolyl)-4,5-dihydro-1*H*-pyrazol-1-yl)-4-substituted-5-(aryl diazenyl)thiazole (**12a**–**e**)

A mixture of 3-(1*H*-indol-3-yl)-5-(*p*-tolyl)-4,5-dihydro-1*H*-pyrazole-1-carbothioamide (**5**) (0.334 g, 1 mmol) and the appropriate hydrazonoyl halides **8a**–**e** (1 mmol) in dioxane (20 mL) containing TEA (0.5 mL) was refluxed for 4 h (monitored by TLC), allowed to cool and the solid formed was collected, washed with EtOH, dried, and recrystallized from DMF to give **12a**–**e**.

*2-(3-(1H-Indol-3-yl)-5-(p-tolyl)-4,5-dihydro-1H-pyrazol-1-yl)-4-methyl-5-(phenyldiazenyl)thiazole* (**12a**). Red solid, (78% yield); mp 210–212 °C; IR (KBr): *v* 3409 (NH), 1642, 1593 (C=N) cm^−1^; ^1^H-NMR (DMSO-*d*_6_): δ 2.35 (s, 3H, CH_3_), 2.54 (s, 3H, CH_3_), 3.24 (dd, 1H, H_A_, *J* = 17.2, 6.3 Hz), 3.40 (dd, 1H, H_B_, *J* = 17.2, 12.1 Hz), 5.88 (dd, 1H, H_X_, *J* = 12.2, 6.3 Hz), 7.17–8.34 (m, 13H, Ar-H), 8.72 (s, 1H, indole-H_2_), 12.10 (br s, 1H, NH); ^13^C-NMR (DMSO-*d*_6_): δ 12.2, 21.0, 36.3, 68.0, 109.5, 111.1, 114.8, 117.1, 119.8, 121.7, 128.8, 130.2, 130.5, 131.4, 133.2, 138.2, 136.1, 147.8, 150.1, 154.0, 161.2; MS, *m*/*z* (%) 476 (M^+^, 61), 349 (19), 249 (44), 152 (50), 29 (100). Anal. Calcd. for C_28_H_24_N_6_S (476.60): C, 70.56; H, 5.08; N, 17.63; found: C, 70.47; H, 5.01; N, 17.53.

*2-(3-(1H-Indol-3-yl)-5-(p-tolyl)-4,5-dihydro-1H-pyrazol-1-yl)-4-methyl-5-(p-tolyldiazenyl)thiazole* (**12b**). Red solid, (72% yield); mp 193–195 °C; IR (KBr): *v* 3403 (NH), 1642, 1590 (C=N) cm^−1^; ^1^H-NMR (DMSO-*d*_6_): δ 2.18 (s, 3H, CH_3_), 2.35 (s, 3H, CH_3_), 2.57 (s, 3H, CH_3_), 3.05 (dd, 1H, H_A_, *J* = 17.2, 6.3 Hz), 3.57 (dd, 1H, H_B_, *J* = 17.2, 12.1 Hz), 5.84 (dd, 1H, H_X_, *J* = 12.2, 6.3 Hz), 7.12–8.34 (m, 12H, Ar-H), 8.71 (s, 1H, indole-H_2_), 12.09 (br s, 1H, NH); MS, *m*/*z* (%) 490 (M^+^, 2), 368 (100), 255 (26), 147 (40), 105 (37), 91 (41), 55 (40). Anal. Calcd. for C_29_H_26_N_6_S (490.62): C, 70.99; H, 5.34; N, 17.13; found: C, 70.76; H, 5.22; N, 17.05.

*2-(3-(1H-Indol-3-yl)-5-(p-tolyl)-4,5-dihydro-1H-pyrazol-1-yl)-5-((4-chlorophenyl)diazenyl)-4-methyl-thiazole* (**12c**). Red solid, (75% yield); mp 206–208 °C; IR (KBr): *v* 3403 (NH), 1642, 1590 (C=N) cm^−1^; ^1^H-NMR (DMSO-*d*_6_): δ 2.35 (s, 3H, CH_3_), 2.58 (s, 3H, CH_3_), 3.28 (dd, 1H, H_A_, *J* = 17.2, 6.3 Hz), 3.57 (dd, 1H, H_B_, *J* = 17.2, 12.1 Hz), 5.80 (dd, 1H, H_X_, *J* = 12.2, 6.3 Hz), 7.17–8.34 (m, 12H, Ar-H), 8.72 (s, 1H, indole-H_2_), 12.09 (br s, 1H, NH); MS, *m*/*z* (%) 511 (M^+^, 34), 452 (69), 262 (86), 189 (100), 136 (37), 95 (48), 43 (50). Anal. Calcd. for C_28_H_23_ClN_6_S (511.04): C, 65.81; H, 4.54; N, 16.44; found: C, 65.65; H, 4.37; N, 16.30.

*2-(3-(1H-Indol-3-yl)-5-(p-tolyl)-4,5-dihydro-1H-pyrazol-1-yl)-5-((4-bromophenyl)diazenyl)-4-methyl-thiazole* (**12d**). Red solid, (78% yield); mp 186–188 °C; IR (KBr): *v* 3409 (NH), 1641, 1586 (C=N) cm^−1^; ^1^H-NMR (DMSO-*d*_6_): δ 2.35 (s, 3H, CH_3_), 2.58 (s, 3H, CH_3_), 3.23 (dd, 1H, H_A_, *J* = 17.2, 6.3 Hz), 3.56 (dd, 1H, H_B_, *J* = 17.2, 12.1 Hz), 5.78 (dd, 1H, H_X_, *J* = 12.2, 6.3 Hz), 7.17–8.34 (m, 12H, Ar-H), 8.71 (s, 1H, indole-H_2_), 12.09 (br s, 1H, NH); MS, *m*/*z* (%) 556 (M^+^, 12), 370 (37), 248 (60), 235 (100), 91 (48), 55 (36). Anal. Calcd. for C_28_H_23_BrN_6_S (555.49): C, 60.54; H, 4.17; N, 15.13; found: C, 60.48; H, 4.11; N, 15.06.

*2-(3-(1H-Indol-3-yl)-5-(p-tolyl)-4,5-dihydro-1H-pyrazol-1-yl)-4-phenyl-5-(phenyldiazenyl)thiazole* (**12e**). Red solid, (72% yield); mp 232–234 °C; IR (KBr): *v* 3404 (NH), 1640, 1591 (C=N) cm^−1^; ^1^H-NMR (DMSO-*d*_6_): δ 2.35 (s, 3H, CH_3_), 3.25 (dd, 1H, H_A_, *J* = 17.2, 6.3 Hz), 3.48 (dd, 1H, H_B_, *J* = 17.2, 12.1 Hz), 5.84 (dd, 1H, H_X_, *J* = 12.2, 6.3 Hz), 7.17–8.34 (m, 18H, Ar-H), 8.72 (s, 1H, indole-H_2_), 12.08 (br s, 1H, NH); ^13^C-NMR (DMSO-*d*_6_): δ 21.0, 35.8, 68.0, 106.0, 109.5, 110.9, 117.3, 119.8, 121.4, 121.2, 125.4, 128.1, 128.4, 129.5, 130.1, 130.4, 131.4, 133.0, 133.4, 135.1, 136.0, 136.4, 147.8, 150.6, 154.6, 168.1; MS, *m*/*z* (%) 538 (M^+^, 9), 451 (4), 432 (43), 326 (62), 225 (100), 77 (64). Anal. Calcd for C_33_H_26_N_6_S (538.66): C, 73.58; H, 4.87; N, 15.60; found: C, 73.49; H, 4.79; N, 15.47.

#### 3.1.4. Alternate Synthesis of **12e**

Synthesis of 2-(3-(1*H*-indol-3-yl)-5-(*p*-tolyl)-4,5-dihydro-1*H*-pyrazol-1-yl)-4-phenylthiazole (**15e**). A mixture of 3-(1*H*-indol-3-yl)-5-(*p*-tolyl)-4,5-dihydro-1*H*-pyrazole-1-carbothioamide (**5**) (0.668 g, 2 mmol) and phenacyl bromide (**14**) (0.398 g, 2 mmol) in absolute EtOH (30 mL) was refluxed for 4 h. The product started to separate out during the course of reaction. The crystalline solid was filtered, washed with water, dried, and recrystallized from EtOH to give pure thiazole **15e** as yellow crystals in 77% yield; mp 177–179 °C; IR (KBr) *υ* 3389 (CH), 1616 (C=N) cm^−1^; ^1^H-NMR (300 MHz, DMSO-*d*_6_): δ 2.34 (s, 3H, CH_3_), 3.17 (dd, 1H, H_A_, *J* = 17.6, 6.1 Hz), 4.19 (dd, 1H, H_B_, *J* = 17.6, 12.2 Hz), 5.80 (dd, 1H, H_X_, *J* = 12.4, 6.1 Hz), 6.85 (s, 1H, thiazole-H5), 7.25–8.32 (m, 13H, Ar-H ), 8.72 (s, 1H, indole-H_2_), 12.10 (br s, 1H, NH); MS, *m*/*z* (%) 434 (M^+^, 2), 324 (37), 225 (100), 183 (68), 157 (74), 72 (58). Anal. Calcd. for C_27_H_22_N_4_S (434.56): C, 74.63; H, 5.10; N, 12.89; found: C, 74.59; H, 5.07; N, 12.69.

##### Coupling of Thiazole **15e** with Benzenediazonium Chloride

To a solution of **15e** (0.434 g, 1 mmol) in EtOH (20 mL) was added sodium acetate trihydrate (0.138 g, 1 mmol), and the mixture was cooled to 0–5 °C in an ice bath. To the resulting cold solution was added portionwise a cold solution of benzenediazonium chloride (prepared by diazotizing aniline (1 mmol) dissolved in hydrochloric acid (6 M, 1 mL) with a solution of sodium nitrite (0.07 g, 1 mmol) in water (2 mL)). After complete addition of the diazonium salt, the reaction mixture was stirred for a further 30 min in an ice bath. The solid that separated was filtered off, washed with water, and finally recrystallized from DMF to give a product that proved identical in all respects (mp, mixed mp and IR spectra) with compound **12e** obtained from reaction of **5** with **8e** in 70% yield**.**

#### 3.1.5. General Procedure for the Reaction of Hydrazonoyl Halides **8** with Thiones **7a**,**b**

To a solution of thione **7a** or **7b** (1 mmol) and the appropriate hydrazonoyl halides **8** (1 mmol) in dioxane (20 mL) was added TEA (0.14 mL, 1 mmol). The reaction mixture was refluxed until all of the starting materials had disappeared (8–12 h, monitored by TLC). The solvent was evaporated and the residue was triturated with MeOH. The solid formed was collected and recrystallized from the appropriate solvent to give products **19a**–**l**. The products **19a**–**l** together with their physical constants are listed below.

*9-Acetyl-2-(1H-indol-3-yl)-7-phenyl-4-(p-tolyl)pyrido[3,2-e][1,2,4]triazolo[4,3-a]pyrimidin-5 (7H)-one* (**19a**). Yellow solid, (80% yield), mp 253–255 °C; IR (KBr): *v* 3435 (NH), 1706, 1633 (2C=O), 1590 (C=N) cm^−1^; ^1^H-NMR (DMSO-*d*_6_): δ 2.35 (s, 3H, CH_3_), 2.49 (s, 3H, CH_3_), 7.30–8.38 (m, 14H, Ar-H and pyridine-H), 8.72 (s, 1H, indole-H_2_), 12.02 (br s, 1H, NH); ^13^C-NMR (DMSO-*d*_6_) *δ*: 21.0, 25.5, 109.4, 113.6, 115.8, 119.0, 120.9, 121.0, 123.9, 126.2, 127.1, 129.2, 129.6, 131.4, 131.8, 133.8, 136.4, 140.9, 146.2, 148.4, 161.0, 163.9, 167.1, 180.6; MS, *m*/*z* (%) 510 (M^+^, 36), 407 (23), 334 (22), 233 (27), 105 (100), 77 (22). Anal. Calcd. for C_31_H_22_N_6_O_2_ (510.55): C, 72.93; H, 4.34; N, 16.46. Found: C, 72.76; H, 4.32; N, 16.28.

*9-Acetyl-2-(1H-indol-3-yl)-4,7-di-p-tolylpyrido[3,2-e][1,2,4]triazolo[4,3-a]pyrimidin-5(7H)-one* (**19b**). Yellow solid, (76% yield), mp 242–244 °C; IR (KBr): *v* 3421 (NH), 1707, 1642 (2C=O), 1589 (C=N) cm^−1^; ^1^H-NMR (DMSO-*d*_6_): δ 2.23 (s, 3H, CH_3_), 2.34 (s, 3H, CH_3_), 2.48 (s, 3H, CH_3_), 7.07-8.28 (m, 13H, Ar-H and pyridine-H), 8.70 (s, 1H, indole-H_2_), 12.03 (br s, 1H, NH); MS, *m*/*z* (%) 524 (M^+^, 22), 509 (100), 381 (32), 231 (96), 173 (79), 55 (63). Anal. Calcd. for C_32_H_24_N_6_O_2_ (524.57): C, 73.27; H, 4.61; N, 16.02. Found: C, 73.12; H, 4.48; N, 15.93.

*9-Acetyl-7-(4-chlorophenyl)-2-(1H-indol-3-yl)-4-(p-tolyl)pyrido[3,2-e][1,2,4]triazolo[4,3-a]pyrimidin-5(7H)-one* (**19c**). Yellow solid, (83% yield), mp 264–265 °C; IR (KBr): *v* 3386 (NH), 1710, 1644 (2C=O), 1589 (C=N) cm^−1^; ^1^H-NMR (DMSO-*d*_6_): δ 2.35 (s, 3H, CH_3_), 2.44 (s, 3H, CH_3_), 7.09–8.34 (m, 13H, Ar-H and pyridine-H), 8.71 (s, 1H, indole-H_2_), 12.09 (br s, 1H, NH); MS, *m*/*z* (%) 545 (M^+^, 3), 490 (24), 258 (30), 152 (47), 29 (100). Anal. Calcd. for C_31_H_21_ClN_6_O_2_ (544.99): C, 68.32; H, 3.88; N, 15.42. Found: C, 68.20; H, 3.69; N, 15.31.

*9-Acetyl-7-(4-bromophenyl)-2-(1H-indol-3-yl)-4-(p-tolyl)pyrido[3,2-e][1,2,4]triazolo[4,3-a]pyrimidin-5(7H)-one* (**19d**). Yellow solid, (78% yield), mp 254–256 °C; IR (KBr): *v* 3389 (NH), 1711, 1643 (2C=O), 1583 (C=N) cm^−1^; ^1^H-NMR (DMSO-*d*_6_): δ 2.35 (s, 3H, CH_3_), 2.45 (s, 3H, CH_3_), 7.08–8.34 (m, 13H, Ar-H and pyridine-H), 8.73 (s, 1H, indole-H_2_), 12.10 (br s, 1H, NH); MS, *m*/*z* (%) 589 (M^+^, 4), 397 (41), 279 (100), 236 (52), 193 (52), 43 (40). Anal. Calcd. for C_31_H_21_BrN_6_O_2_ (589.44): C, 63.17; H, 3.59; N, 14.26. Found: C, 63.09; H, 3.42; N, 14.17.

*Ethyl2-(1H-Indol-3-yl)-5-oxo-7-phenyl-4-(p-tolyl)-5,7-dihydropyrido[3,2-e][1,2,4]triazolo[4,3-a]-pyrimidine-9-carboxylate* (**19e**). Yellow solid, (75% yield), mp 212–214 °C; IR (KBr): *v* 3342 (NH), 1718, 1644 (2C=O), 1579 (C=N) cm^−1^; ^1^H-NMR (DMSO-*d*_6_): δ 1.29 (t, *J* = 7.1 Hz, 3H, CH_3_), 2.34 (s, 3H, CH_3_), 4.29 (q, *J* = 7.1 Hz, 2H, CH_2_), 7.13–8.30 (m, 14H, Ar-H and pyridine-H), 8.73 (s, 1H, indole-H_2_), 12.12 (br s, 1H, NH); MS, *m*/*z* (%) 540 (M^+^, 2), 521 (16), 361 (13), 270 (69),167 (38), 91 (100). Anal. Calcd. for C_32_H_24_N_6_O_3_ (540.57): C, 71.10; H, 4.47; N, 15.55. Found: C, 71.07; H, 4.39; N, 15.37.

*Ethyl2-(1H-Indol-3-yl)-5-oxo-4,7-di-p-tolyl-5,7-dihydropyrido[3,2-e][1,2,4]triazolo[4,3-a]pyrimidine-9-carboxylate* (**19f**). Yellow solid, (77% yield), mp 238–240 °C; IR (KBr): *v* 3348 (NH), 1746, 1673 (2C=O), 1576 (C=N) cm^−1^; ^1^H-NMR (DMSO-*d*_6_): δ 1.30 (t, *J* = 7.1 Hz, 3H, CH_3_), 2.23 (s, 3H, CH_3_), 2.35 (s, 3H, CH_3_), 4.26 (q, *J* = 7.1 Hz, 2H, CH_2_), 7.08–8.32 (m, 13H, Ar-H and pyridine-H), 8.72 (s, 1H, indole-H_2_), 12.09 (br s, 1H, NH); MS, *m*/*z* (%) 554 (M^+^, 4), 522 (24), 431 (100), 326 (88), 282 (25), 91 (10). Anal. Calcd. for C_33_H_26_N_6_O_3_ (554.60): C, 71.47; H, 4.73; N, 15.15. Found: C, 71.38; H, 4.70; N, 15.04.

*Ethyl2-(1H-Indol-3-yl)-7-(4-methoxyphenyl)-5-oxo-4-(p-tolyl)-5,7-dihydropyrido[3,2-e][1,2,4]triazolo-[4,3-a]pyrimidine-9-carboxylate* (**19g**). Yellow solid, (70% yield), mp 193–195 °C; IR (KBr): *v* 3390 (NH), 1740, 1674 (2C=O), 1578 (C=N) cm^−1^; ^1^H-NMR (DMSO-*d*_6_): δ 1.32 (t, *J* = 7.1 Hz, 3H, CH_3_), 2.35 (s, 3H, CH_3_), 3.82 (s, 3H, OCH_3_), 4.34 (q, *J* = 7.1 Hz, 2H, CH_2_), 7.03–8.34 (m, 13H, Ar-H and pyridine-H), 8.72 (s, 1H, indole-H_2_), 12.10 (br s, 1H, NH); MS, *m*/*z* (%) 570 (M^+^, 10), 354 (52), 311 (62), 267 (85), 72 (54), 59 (100). Anal. Calcd. for C_33_H_26_N_6_O_4_ (570.60): C, 69.46; H, 4.59; N, 14.73. Found: C, 69.39; H, 4.48; N, 14.59.

*Ethyl7-(4-Chlorophenyl)-2-(1H-indol-3-yl)-5-oxo-4-(p-tolyl)-5,7-dihydropyrido[3,2-e][1,2,4]triazolo-[4,3-a]pyrimidine-9-carboxylate* (**19h**). Yellow solid, (78% yield), mp 243–245 °C; IR (KBr): *v* 3383 (NH), 1742, 1675 (2C=O), 1577 (C=N) cm^−1^; ^1^H-NMR (DMSO-*d*_6_): δ 1.31 (t, *J* = 7.1 Hz, 3H, CH_3_), 2.35 (s, 3H, CH_3_), 4.37 (q, *J* = 7.1 Hz, 2H, CH_2_), 7.07-8.34 (m, 13H, Ar-H and pyridine-H), 8.72 (s, 1H, indole-H_2_), 12.09 (br s, 1H, NH); MS, *m*/*z* (%) 575 (M^+^, 17), 352 (12), 293 (29), 126 (23), 72 (59), 59 (100). Anal. Calcd. for C_32_H_23_ClN_6_O_3_ (575.02): C, 66.84; H, 4.03; N, 14.62. Found: C, 66.69; H, 4.01; N, 14.47.

*2-(1H-Indol-3-yl)-5-oxo-N,7-diphenyl-4-(p-tolyl)-5,7-dihydropyrido[3,2-e][1,2,4]triazolo[4,3-a]-pyrimidine-9-carboxamide* (**19i**). Yellow solid, (82% yield), mp 268–270 °C; IR (KBr): *v* 3385, 3213 (2NH), 1679, 1641 (2C=O), 1598 (C=N) cm^−1^; ^1^H-NMR (DMSO-*d*_6_): δ 2.35 (s, 3H, CH_3_), 7.02–8.34 (m, 19H, Ar-H and pyridine-H), 8.72 (s, 1H, indole-H_2_), 10.92 (br s, 1H, NH), 12.10 (br s, 1H, NH); MS, *m*/*z* (%) 587 (M^+^, 2), 441 (30), 271 (43), 158 (100), 128 (43), 91 (31). Anal. Calcd. for C_36_H_25_N_7_O_2_ (587.63): C, 73.58; H, 4.29; N, 16.69. Found: C, 73.52; H, 4.15; N, 16.57.

*7-(4-Chlorophenyl)-2-(1H-indol-3-yl)-5-oxo-N-phenyl-4-(p-tolyl)-5,7-dihydropyrido[3,2-e][1,2,4]-triazolo[4,3-a]pyrimidine-9-carboxamide* (**19j**). Yellow solid, (80% yield), mp 279–281 °C; IR (KBr): *v* 3387, 3198 (2NH), 1683, 1642 (2C=O), 1597 (C=N) cm^−1^; ^1^H-NMR (DMSO-*d*_6_): δ 2.34 (s, 3H, CH_3_), 7.02–8.34 (m, 18H, Ar-H and pyridine-H), 8.72 (s, 1H, indole-H_2_), 11.13 (br s, 1H, NH), 12.10 (br s, 1H, NH); MS, *m*/*z* (%) 622 (M^+^, 1), 341 (38), 267 (100), 129 (35), 98 (52), 57 (63). Anal. Calcd. for C_36_H_24_ClN_7_O_2_ (622.07): C, 69.51; H, 3.89; N, 15.76. Found: C, 69.42; H, 3.81; N, 15.59.

*9-Acetyl-4-(4-chlorophenyl)-2-(1H-indol-2-yl)-7-phenylpyrido[3,2-e][1,2,4]triazolo[4,3-a]pyrimidin-5(7H)-one* (**19k**). Yellow solid, (78% yield), mp 271–273 °C; IR (KBr): *v* 3427 (NH), 1701, 1674 (2C=O), 1589 (C=N) cm^−1^; ^1^H-NMR (DMSO-*d*_6_): δ 2.49 (s, 3H, CH_3_), 7.21–8.10 (m, 14H, Ar-H and pyridine-H), 8.73 (s, 1H, indole-H_2_), 12.04 (br s, 1H, NH); MS, *m*/*z* (%) 530 (M^+^, 100), 515 (53), 219 (47), 147 (87), 97 (21), 57 (79). Anal. Calcd. for C_30_H_19_ClN_6_O_2_ (530.96): C, 67.86; H, 3.61; N, 15.83. Found: C, 67.69; H, 3.54; N, 15.71.

*9-Acetyl-4-(4-chlorophenyl)-2-(1H-indol-2-yl)-7-(*p*-tolyl)pyrido[3,2-e][1,2,4]triazolo [4,3-a]pyrimidin-5(7H)-one* (**19l**). Yellow solid, (78% yield), mp 252–254 °C; IR (KBr): *v* 3403 (NH), 1705, 1671 (2C=O), 1583 (C=N) cm^−1^; ^1^H-NMR (DMSO-*d*_6_): δ 2.29 (s, 3H, CH_3_), 2.46 (s, 3H, CH_3_), 7.09–8.34 (m, 13H, Ar-H and pyridine-H), 8.73 (s, 1H, indole-H_2_), 12.10 (br s, 1H, NH); MS, *m*/*z* (%) 544 (M^+^, 3), 362 (15), 303 (100), 227 (27), 117 (39), 55 (21). Anal. Calcd. for C_31_H_21_ClN_6_O_2_(544.99): C, 68.32; H, 3.88; N, 15.42. Found: C, 68.25; H, 3.69; N, 15.31.

#### 3.1.6. Alternate Synthesis of **19a**,**e**,**i**

Equimolar amounts of 1-(1*H*-indol-3-yl)-3-(*p*-tolyl)prop-2-en-1-one (**3a**) (0.261 g, 1 mmol) and 7-amino-1-phenyl-[1,2,4]triazolo[4,3-*a*]pyrimidin-5(1*H*)-one derivatives **20a**,**e**,**i** (1 mmol) in acetic acid (15 mL), was refluxed for 10 h then cooled to room temperature. The solid precipitated was collected, washed with water, dried, and recrystallized from DMF to give the corresponding products, **19a**,**e**,**i** which were identical in all respects (mp, mixed mp and IR spectra) with those obtained from reaction of thione **7a** with hydrazonoyl chlorides **8a**,**e**,**i** but the % yields are 69%, 67%, and 70%, respectively.

#### 3.1.7. Synthesis of *N*’-(1-(1*H*-Indol-3-yl)ethylidene)-2-cyanoacetohydrazide (**22**)

To a solution of 2-cyanoacetohydrazide (**21**) (1.0 g, 10 mmol and 3-acetyl-1*H*-indole (**1**) (0.159 g, 1 mmol) in ethanol (30 mL), acetic acid (2 mL) was added. The reaction mixture was heated under reflux for 3 h then left to cool. The solid product formed was collected by filtration, dried, and then crystallized from the appropriate solvent as yellow solid, (76% yield); mp 231–233 °C; IR (KBr): *v* 3402 (NH), 2225 (CN) 1670 (C=O), 1602 (C=N) cm^−1^; ^1^H-NMR (DMSO-*d*_6_): δ 2.57 (s, 3H, CH_3_), 3.58 (s, 2H, CH_2_), 7.13–7.23 (m, 2H, Ar-H), 7.47 (d, *J* = 6.9 Hz, 1H, Ar-H), 8.18 (d, *J* = 6.9 Hz, 1H, Ar-H), 8.29 (s, 1H, indole-H_2_), 10.42 (br s, 1H, NH), 11.88 (br s, 1H, NH); MS, *m*/*z* (%) 240 (M^+^, 17), 170 (100), 153 (56), 183 (8), 125 (11), 70 (19), 55 (26). Anal. Calcd. for C_13_H_12_N_4_O (240.26): C, 64.99; H, 5.03; N, 23.32. Found C, 64.85; H, 5.01; N, 23.22.

#### 3.1.8. Synthesis of 3-(2-(1-(1*H*-Indol-3-yl)ethylidene)hydrazinyl)-2-cyano-3-oxo-*N*-phenylpropane thioamide (**25**)

To a stirred solution of potassium hydroxide (0.56 g, 10 mmol) in DMF (30 mL) was added compound **22** (2.40 g, 10 mmol). After stirring for 30 minutes, phenyl isothiocyanate (**23**) (1.35 g, 10 mmol) was added to the resulting mixture. Stirring was continued overnight. The reaction mixture was acidified with HCl and the solid product was filtered off, washed with water, and dried. Recrystallization from EtOH gave pure **25** as yellow solid, (70% yield); mp 143–145 °C; IR (KBr): *v* 3402, 3387, 3182 (3NH), 2230 (CN) 1664 (C=O), 1600 (C=N) cm^−1^; ^1^H-NMR (DMSO-*d*_6_): δ 2.57 (s, 3H, CH_3_), 7.14–7.23 (m, 2H, Ar-H), 7.46 (d, *J* = 6.9 Hz, 1H, Ar-H), 8.18 (d, *J* = 6.9 Hz, 1H, Ar-H), 8.29 (s, 1H, indole-H_2_), 9.85 (br s, 1H, NH), 10.40 (br s, 1H, NH), 11.88 (br s, 1H, NH), 13.11 (s, 1H, SH); MS, *m*/*z* (%) 375 (M^+^, 56), 207 (83), 165 (100), 119 (32), 77 (20). Anal. Calcd. for C_20_H_17_N_5_OS (375.45): C, 63.98; H, 4.56; N, 18.65. Found C, 63.78; H, 4.48; N, 18.47.

#### 3.1.9. Reaction of **25** with Hydrazonoyl Chlorides **8a**,**e**,**i**

A mixture of **25** (0.375 g, 1 mmol) and *N*’-phenylbenzohydrazonoyl chloride **8a**,**e**,**i** (1mmol) in dioxane (30 mL) containing TEA (0.7 mL) was refluxed for 5 h (monitored by TLC), allowed to cool and the solid formed was collected, washed with EtOH, dried, and recrystallized from DMF to give the respective 1,3,4-thiadiazole **27a**–**c**.

*N’-(1-(1H-Indol-3-yl)ethylidene)-2-(5-acetyl-3-phenyl-1,3,4-thiadiazol-2(3H)-ylidene)-2-cyanoaceto-hydrazide* (**27a**). Yellow solid, (68% yield); mp 191–193 °C; IR (KBr): *v* 3425, 3385 (2NH), 2227 (CN), 1695, 1664 (2C=O), 1608 (C=N) cm^−1^; ^1^H-NMR (DMSO-*d*_6_): δ 2.47 (s, 3H, CH_3_), 2.58 (s, 3H, CH_3_), 7.19–8.25 (m, 9H, Ar-H), 8.68 (s, 1H, indole-H_2_), 10.89 (br s, 1H, NH), 11.93 (br s, 1H, NH); ^13^C-NMR (DMSO-*d*_6_) δ: 14.0, 24.5, 74.0, 116.4, 110.0, 116.7, 118.7, 121.4, 124.8, 128.4, 136.3, 130.5, 135.0, 143.4, 148.4, 157.4, 164.0, 189.9; MS, *m*/*z* (%) 442 (M^+^, 16), 364 (39), 275 (52), 215 (86), 107 (100), 81 (41), 43 (37). Anal. Calcd. for C_23_H_18_N_6_O_2_S (442.49): C, 62.43; H, 4.10; N, 18.99. Found C, 62.29; H, 4.03; N, 18.79.

*Ethyl5-(2-(2-(1-(1H-Indol-3-yl)ethylidene)hydrazinyl)-1-cyano-2-oxoethylidene)-4-phenyl-4,5-dihydro-1,3,4-thiadiazole-2-carboxylate* (**27b**). Yellow solid, (68% yield); mp 191–193 °C; IR (KBr): *v* 3425, 3385 (2NH), 2227 (CN), 1695, 1664 (2C=O), 1608 (C=N) cm^−1^; ^1^H-NMR (DMSO-*d*_6_): δ 1.30 (t, *J* = 7.1 Hz, 3H, CH_3_), 2.55 (s, 3H, CH_3_), 4.27 (q, *J* = 7.1 Hz, 2H, CH_2_), 7.10–8.29 (m, 9H, Ar-H), 8.69 (s, 1H, indole-H_2_), 10.88 (br s, 1H, NH), 11.97 (br s, 1H, NH); ^13^C-NMR (DMSO-*d*_6_) δ: MS, 14.0, 24.5, 74.0, 110.0, 110.5, 118.9, 120.4, 121.5, 125.1, 130.3, 130.5, 133.0, 143.4, 148.5, 157.0, 164.0, 189.8; *m/z* (%) 472 (M^+^, 15), 412 (37), 250 (43), 139 (100), 108 (29), 43 (40). Anal. Calcd. for C_24_H_20_N_6_O_3_S (472.52): C, 61.00; H, 4.27; N, 17.79. Found C, 61.07; H, 4.17; N, 17.64.

*5-(2-(2-(1-(1H-Indol-3-yl)ethylidene)hydrazinyl)-1-cyano-2-oxoethylidene)-N,4-diphenyl-4,5-dihydro-1,3,4-thiadiazole-2-carboxamide* (**27c**). Yellow solid, (67% yield); mp 276–278 °C; IR (KBr): *v* 3417, 3363, 3197 (3NH), 2230 (CN), 1674, 1627 (2C=O), 1601 (C=N) cm^−1^; ^1^H-NMR (DMSO-*d*_6_) δ: 2.55 (s, 3H, CH_3_), 7.06–8.28 (m, 14H, Ar-H), 8.47 (s, 1H, indole-H_2_), 9.87 (br s, 1H, NH), 10.66 (br s, 1H, NH), 11.49 (br s, 1H, NH); MS, *m*/*z* (%) 519 (M^+^, 100), 372 (53), 266 (38), 137 (29), 120 (57), 43 (40). Anal. Calcd. for C_28_H_21_N_7_O_2_S (519.58): C, 64.73; H, 4.07; N, 18.87. Found C, 64.58; H, 4.03; N, 18.72.

### 3.2. Antitumor Activity Assay

The tested human carcinoma cell lines were obtained from the American Type Culture Collection (ATCC, Rockville, MD, USA). The cells were grown on RPMI-1640 medium supplemented with 10% heat inactivated fetal calf serum, 1% l-glutamine, and 50 µg/mL gentamycin. The cells were maintained at 37 °C in a humidified atmosphere with 5% CO_2_ incubator (Shel lab 2406, New York**,** NY, USA) and were sub-cultured two to three times a week. For antitumor assays, the tumor cell lines were suspended in medium at concentration 5 × 10^4^ cell/well in Corning^®^ 96-well tissue culture plates, then incubated for 24 h. The tested compounds were then added into 96-well plates (six replicates) to achieve eight concentrations for each compound (started from 200 to 1.56 µg/mL). Six vehicle controls with media or 0.1% DMSO were run for each 96-well plate as a control. After incubating for 24 h, the numbers of viable cells were determined by the MTT assay. Briefly, the media was removed from the 96-well plate and replaced with 100 µL of fresh culture RPMI 1640 medium without phenol red then 10 µL of the 12 mM MTT (3-[4,5-dimethylthiazol- 2-yl]-2,5-diphenyltetrazolium bromide (MTT; Sigma Chemical Co., St. Louis, MO, USA) stock solution (5 mg of MTT in 1 mL of PBS) to each well including the untreated controls. The 96-well plates were then incubated at 37 °C and 5% CO_2_ for 4 h. An 85 µL aliquot of the media was removed from the wells, and 50 µL of DMSO was added to each well and mixed thoroughly with the pipette and incubated at 37 °C for 10 min. Then, the optical density was measured at 590 nm with the microplate reader ((SunRise, TECAN, Inc, Männedorf, Switzerland) to determine the number of viable cells and the percentage of viability was calculated as [1 − (ODt/ODc)] × 100%, where ODt is the mean optical density of wells treated with the tested sample and ODc is the mean optical density of untreated cells. The relation between surviving cells and drug concentration is plotted to get the survival curve of each tumor cell line after treatment with the specified compound. The 50% inhibitory concentration (IC_50_), the concentration required to cause toxic effects in 50% of intact cells, was estimated from graphic plots of the dose response curve for each concentration using Graphpad Prism software (San Diego, CA, USA) [47,48].

## 4. Conclusions

3-Acetylindole proved to be a useful precursor for synthesis of various 1,3-thiazoles, 1,2,4-thiadiazoles and pyrido[3,2-*e*][1,2,4]triazolo[4,3-*a*]pyrimidin-5(7*H*)-one. The structures of the newly synthesized compounds were confirmed by spectral data and elemental analyses. Some of the new compounds were tested in vitro against the MCF-7 human breast carcinoma cell line and compared with doxorubicin as the standard, using the MTT viability assay. Most of the tested compounds were found to have moderate to high anticancer activity.

## Figures and Tables

**Table 1:**
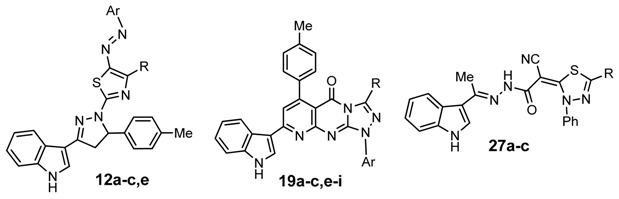
The antitumor activities of the tested compounds compared with reference doxorubicin evaluated using MTT assay on the MCF-7 breast cancer cell line.

Compound No.	R	Ar	IC_50_ (µM)
**12a**	CH_3_	Ph	4.92
**12b**	CH_3_	4-MeC_6_H_4_	0.95
**12c**	CH_3_	4-ClC_6_H_4_	14.52
**12e**	Ph	Ph	19.44
**19a**	CH_3_CO	Ph	6.88
**19b**	CH_3_CO	4-MeC_6_H_4_	4.68
**19c**	CH_3_CO	4-ClC_6_H_4_	69.85
**19e**	CH_3_CH_2_OCO	Ph	4.83
**19f**	CH_3_CH_2_OCO	4-MeC_6_H_4_	5.49
**19g**	CH_3_CH_2_OCO	4-MeOC_6_H_4_	3.05
**19h**	CH_3_CH_2_OCO	4-ClC_6_H_4_	18.61
**19i**	PhNHCO	Ph	6.07
**27a**	CH_3_CO	Ph	2.04
**27b**	CH_3_CH_2_OCO	Ph	1.01
**27c**	PhNHCO	Ph	1.27
Doxorubicin	-	-	0.75
